# Time to Update and Quantitative Changes in the Results of Cochrane Pregnancy and Childbirth Reviews

**DOI:** 10.1371/journal.pone.0011553

**Published:** 2010-07-13

**Authors:** Wanlop Jaidee, David Moher, Malinee Laopaiboon

**Affiliations:** 1 Department of Public Health Foundation, Faculty of Public Health, Burapha University, Chonburi, Thailand; 2 Ottawa Methods Centre, Clinical Epidemiology Program, Ottawa Hospital Research Institute, Ottawa, Ontario, Canada; 3 Department of Epidemiology and Community Medicine, Faculty of Medicine, University of Ottawa, Ottawa, Ontario, Canada; 4 Department of Biostatistics and Demography, Faculty of Public Health, Khon Kaen University, Khon Kaen, Thailand; Johns Hopkins Bloomberg School of Public Health, United States of America

## Abstract

**Background:**

The recommended interval between updates for systematic reviews included in The Cochrane Library is 2 years. However, it is unclear whether this interval is always appropriate. Whereas excessive updating wastes time and resources, insufficient updating allows out-of-date or incomplete evidence to guide clinical decision-making. We set out to determine, for Cochrane pregnancy and childbirth reviews, the frequency of updates, factors associated with updating, and whether updating frequency was appropriate.

**Methodology/Principal Findings:**

Cochrane pregnancy and childbirth reviews published in Issue 3, 2007 of the Cochrane Database of Systematic Reviews were retrieved, and data were collected from their original and updated versions. Quantitative changes were determined for one of the primary outcomes (mortality, or the outcome of greatest clinical significance). Potential factors associated with time to update were assessed using the Cox proportional hazard model. Among the 101 reviews in our final sample, the median time before the first update was 3.3 years (95% CI 2.7–3.8). Only 32.7% had been updated within the recommended interval of 2 years. In 75.3% (76/101), a median of 3 new trials with a median of 576 additional participants were included in the updated versions. There were quantitative changes in 71% of the reviews that included new trials (54/76): the median change in effect size was 18.2%, and the median change in 95% CI width was 30.8%. Statistical significance changed in 18.5% (10/54) of these reviews, but conclusions were revised in only 3.7% (2/54). A shorter time to update was associated with the same original review team at updating.

**Conclusions/Significance:**

Most reviews were updated less frequently than recommended by Cochrane policy, but few updates had revised conclusions. Prescribed time to update should be reconsidered to support improved decision-making while making efficient use of limited resources.

## Introduction

Systematic reviews have become increasingly popular in recent years [1] as the best source of evidence for health care practitioners and others. The scientific process used to produce a systematic review has put these reviews at the centre of health care systems in many countries. At their core systematic reviews aggregate evidence from primary studies, globally. Clinicians can use these reviews to guide patient care and develop clinical practice guidelines. Likewise, decision makers use reviews to help make informed (and evidence based) decisions at all levels of the health care system [2].

When information about an intervention is dynamic and changes over time [3], systematic reviews provide an important source of up-to-date information to support clinical decision-making [4]. Systematic reviews are less useful if they are not up to date. Recent studies have reported that 37% to 70% of systematic reviews in The Cochrane Library have been updated [5], [6]. Of the Cochrane reviews (CRs) that are updated, only a small proportion (3% to 9%) lead to changes to results and conclusions [5], [7]–[9]. Some updates result in increased precision and statistical significance of the primary outcomes; in others, the reverse effect occurs. The 2-year updating policy of The Cochrane Collaboration [10] might not be appropriate to all CRs. Frequent updates to CRs might be unnecessary and waste resources; on the other hand, less frequent updates could render the results of CRs outdated, misleading, or both [4].

A recent analysis by Shojania and colleagues [11] of a sample of systematic reviews showed that the median duration of survival before the need for an update was signaled was 5.5 years (95% CI 4.36–7.67). A signal that the evidence was out of date occurred within 2 years for 23% of reviews and within 1 year for 15% of reviews. Shorter survival times were seen in reviews that addressed cardiovascular topics. For the purposes of our analysis, updates were deemed to be warranted if they showed a 50% or greater change in quantitative results, including a change in statistical significance and a relative change in the magnitude of effect. The systematic reviews in the Cochrane Database of Systematic Reviews cover many clinical areas, and the ideal interval for updating may vary from one area to another, depending on factors such as the number of new trials and participants, search strategies and databases, and the time to publication of new trials [4].

The Cochrane Pregnancy and Childbirth Group (PCG) was, in October 1992, the first to register with the Cochrane Collaboration [12]. The number of PCG reviews and protocols has been increasing continuously since that time [13], [14]. The survival time of reviews before they are updated, potential factors that trigger updates and quantitative criteria for updating might differ from those in other clinical areas. The purpose of this study was to identify the time to update and to describe the status of updated PCG reviews. Changes in effect size, confidence interval (CI) width, and statistical significance were quantified to determine whether they were of sufficient importance to warrant the update. Potential factors associated with updating time were also assessed.

## Methods

One investigator (WJ) searched for the PCG reviews in the Cochrane Library issue3, 2007 using the query topic “Pregnancy and Childbirth”. All 381 registered PCG reviews formed the original retrospective cohort.

### Criteria for identifying updated reviews and their original versions

Reviews were identified as updates if their first published version had appeared before 2007 Issue 3 and their latest versions were of this issue. The updated reviews were identified from Archie the Cochrane Collaboration's Information System (IMS) website. In the history page of each review, if the version was not ‘the first published’, it was identified as an updated version. The original versions of reviews identified as updates were then searched from previous Cochrane Library CDs using the digital object identifier (DOI) number.

### Main outcome and predicting factors

The main outcome, time to update, was defined as the duration from the date of first publication to the date of the most recent substantive amendment (update). Both dates were reported on the “cover” sheet of each review.

Factors potentially associated with time to update were clinical topic classification, number of additional trials, number of additional participants, the use of additional databases, new search strategies, author affiliation, and country of origin. PCG topics were classified as follows: (1) antenatal care; (2) pregnancy complications; (3) fetal complications; (4) intrapartum issues; or (5) postpartum issues. Author affiliation of first and corresponding authors was classified as academic (e.g., university-based) or non-academic. The non-academic category included hospitals, medical practices, health research institutes and other organizations such as the World Health Organization. Economic status [15] was used to classify the country of origin as “developed” or “developing.” Changes in the review team were classified as follows: (1) same as original version; (2) included new author(s); (3) changed author(s); or (4) changed new team.

### Quantitative change

Quantitative change was defined as a change in the updated version relative to the results of the original meta-analytic results, in the magnitude and/or direction of effect, statistical significance, relative effect size, or width of the CI. Quantitative change was not measured for reviews which did not contain a meta-analysis, had no or only one additional trial, or generated new comparisons. For reviews with more than 1 primary outcome, the primary mortality outcome or the outcome of greatest clinical significance (e.g., preterm labour, low birth weight, prolongation of pregnancy, etc.) was used. The outcome of greatest clinical significance was selected by a gynaecologist of Khon Kaen university hospital. Specific criteria for quantitative changes were as follows:

1. **Change in effect size.** The effect size was deemed to have changed when the result of the updated meta-analysis showed a relative change in effect size when compared with the result reported in the first published version. The change in effect size was calculated as the ratio of (A) the difference between the updated and the original pooled treatment effect to (B) the original pooled treatment effect. The direction of the change in effect size was also observed.
2. **Change in the width of the effect size CI.** The change in CI width was calculated as the ratio between the difference between (A) the updated and original CI width to (B) the original CI width.
3. **Change in statistical significance.** This was defined as a change from a statistically significant finding for the primary outcome to a non-statistically significant finding, or the reverse. To eliminate borderline changes in statistical significance, a change from *p* = 0.04 to *p* = 0.06 or from *p* = 0.06 to *p* = 0.04 was not counted as a change in statistical significance.

A quantitative change was detected when at least one criterion was met. The changes were classified into 3 groups: no change; minor changes (at least 1 quantitative change, but with no effect on the conclusion); and major changes (at least 1 quantitative change that affected the conclusion).

### Change in meta-analysis conclusions

The conclusion of the meta-analysis was considered to have changed when the interpretation of findings in the updated review was substantially altered from the interpretation of the original findings. A change in style or wording that did not alter the substance or meaning of the conclusion was not considered a change in the conclusion [5], [16].

### Data extraction

Data on the main outcome and other characteristics were collected (WJ) from the original systematic review and its associated updates. These data included author affiliations (including country), issue of publication, date of most recent substantive amendment, update frequency, search strategies and search resources, number of included trials and participants, and summary statistics (e.g., relative risk), including the CIs of the effect sizes of the primary outcomes. These data were extracted using a specially designed data collection form. A second member of the research team (ML), using the same methods, independently collected these data from a random sample of 20 updated reviews; the 2 sets of results were compared for the purpose of validation. Discrepancies, such as differences in primary outcome and changes in conclusion, between the 2 sets of extracted data were resolved by consensus.

### Analysis of time to update and associated factors

Kaplan–Meier survival curves were used to estimate an average time to update and its 95% CI [17]. A Cox proportional hazards model was applied to examine the association between the potential factors and time to update of the cohort of PCG reviews. The effect of each factor is presented as a hazard ratio (HR) and 95% CI. The statistical software Stata, version 10.0 (StataCorp LP, College Station, Tex.), was used to complete the data analyses.

## Results

**Figure 1:**
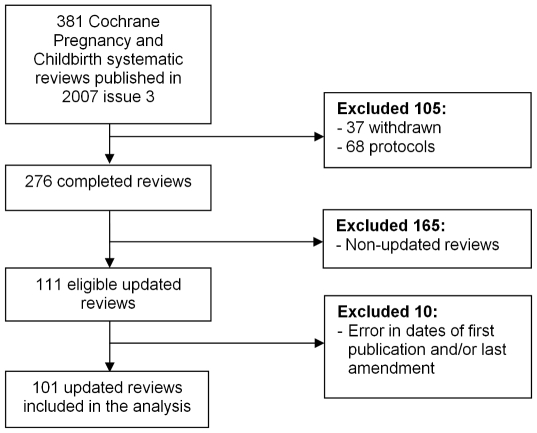
Flow diagram of selection of reviews for analysis.

From the retrospective cohort of 381 PCG reviews we excluded 105: of these, 37 had been withdrawn from the Cochrane database, and 68 were still protocols. There were 276 completed PCG reviews in the 2007 volume, Issue 3. Of these, 111 were updates according to our criteria, i.e. the latest versions of the study cohort were different from those of the first published version. However, examination of the full texts revealed errors in 10 reviews in the dates of first publication and/or last amendment. Our analysis was therefore limited to 101 updated reviews (Fig. 1).

### Characteristics of updated PCG reviews

The 101 PCG reviews had been updated 1 to 6 times; most had been updated only once (67.3%, 68/101). A small number of reviews had been updated 2 or more times: (33 reviews had been updated at least twice, 11 reviews had been updated 3 or more times, and 4 reviews had been updated 4 or more times). In the following we present the survival time to update, quantitative changes, and factors associated with time to update for reviews at their first update only.

Among the 101 reviews, the largest proportion of updates was seen in reviews that addressed intrapartum issues (33.7%, 34/101), while 24.8% (25/101) addressed fetal complications and only 5% (5/101) addressed postpartum issues. The majority of the corresponding authors who conducted the reviews were in academic institutions (71.3%, 72/101), and 93.1% (94/101) were in developed countries. From the updates, 29.7% (30/101) included/changed at least one new review author and 3.0% (3/101) had a new review team. In 27.7% (28/101) of the updates, new databases were searched, and 24.8% (25/101) used new search strategies. Most reviews were updated by the addition of new trials (75.3%, 76/101), with a median of 3 trials (IQR 1–5) and a median of 576 new participants (IQR 180–1386). Of these 76 reviews, 71% (54/76) updated the primary outcomes (Table 1). For the 25 updated reviews that did not include additional new trials and participants in the primary outcomes, 40.0% (10/25) added new comparisons, or subgroup analyses.

**Table 1:** Characteristics of updated reviews at first update period (101 reviews).

Characteristic	Number (%)
*Topic classification*	
Antenatal care	17 (16.8)
Fetal complications	25 (24.8)
Intrapartum issues	34 (33.7)
Pregnancy complications	20 (19.8)
Postpartum issues	5 (5.0)
*Author affiliation*	
Academic	72 (71.3)
Non-academic	29 (28.7)
*Author country*	
Developed country	94 (93.1)
Developing country	7 (6.9)
*Review team*	
Same original version	68 (67.3)
Included new author(s)	19 (18.8)
Changed author(s)	11 (10.9)
New review team	3 (3.0)
*Additional new database searched*	28 (27.7)
*New search strategies*	25 (24.8)
*Additional trials included*	76 (75.3)
* Median of included trials* (q1–q3)* = 3 (1–5) trials	
* Median of included participants* (q1–q3)* = 576 (180–1386) participants	
*With quantitative change in primary outcome* *	54 (71.0)
*With change in conclusions* *	2 (2.6)

*For 76 reviews that included new trials.

### Time to the first update

**Figure 2:**
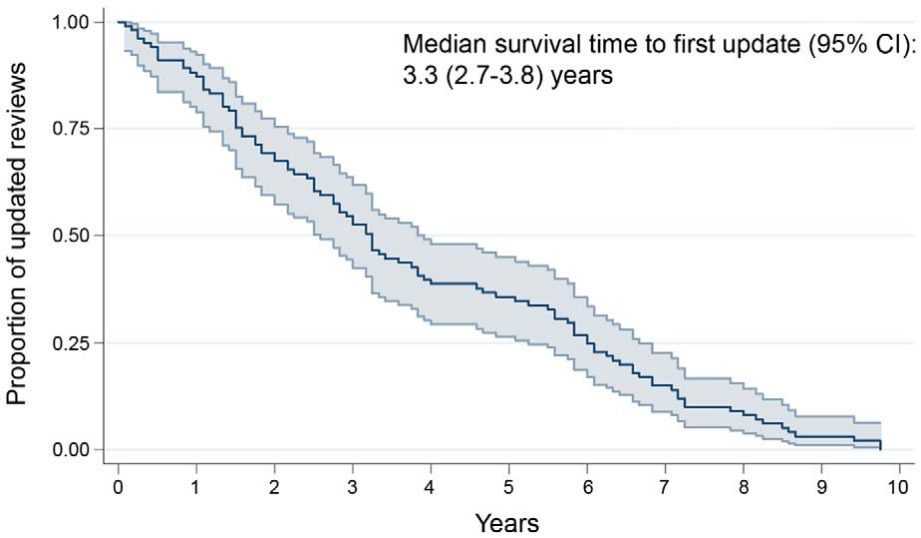
Kaplan–Meier curves with 95% confidence interval for sample of 101 reviews at first update.

The median time to the first update was 3.3 years (95% CI 2.7–3.8) for the 101 updated reviews (Fig. 2). Only 12.9% (13/101) of the reviews were updated within 1 year, and 32.7% (33/101) were updated within 2 years. Intrapartum issues had the fastest time to update, with a median of 2.5 years (95% CI 1.6–3.6), followed by postpartum issues, with a median of 2.8 years (95% CI 2.1–3.6, Table 2).

**Table 2:** Time to first update of the 101 updated reviews, by topic classification and presence or absence of additional trials.

	Overall		With additional trials		Without additional trials	
	Reviews, n	Years to update, median (95% CI)	Reviews, n	Years to update, median (95% CI)	Review, n	Years to update, median (95% CI)
Overall	101	3.3 (2.7–3.8)	76	3.3 (2.5–4.0)	25	2.5 (0.6–4.4)
*CPC topic classification*						
Antenatal care	17	5.5 (2.7–8.3)	14	5.6 (1.4–9.8)	3	0.5, 2.5, 8.6*
Fetal complications	25	3.9 (0.9–6.9)	14	3.9 (2.5–5.3)	11	1.5 (0.5–6.3)
Intrapartum issues	34	2.5 (1.6–3.6)	28	2.8 (1.8–3.7)	6	1.8 (0.5–3.1)
Postpartum issues	5	2.8 (2.1–3.6)	5	2.8 (2.1–3.6)	–	–
Pregnancy complications	20	3.3 (0.6–6.1)	15	3.3 (0.5–6.2)	5	3.3 (2.7–3.8)

*Actual values.

**Figure 3:**
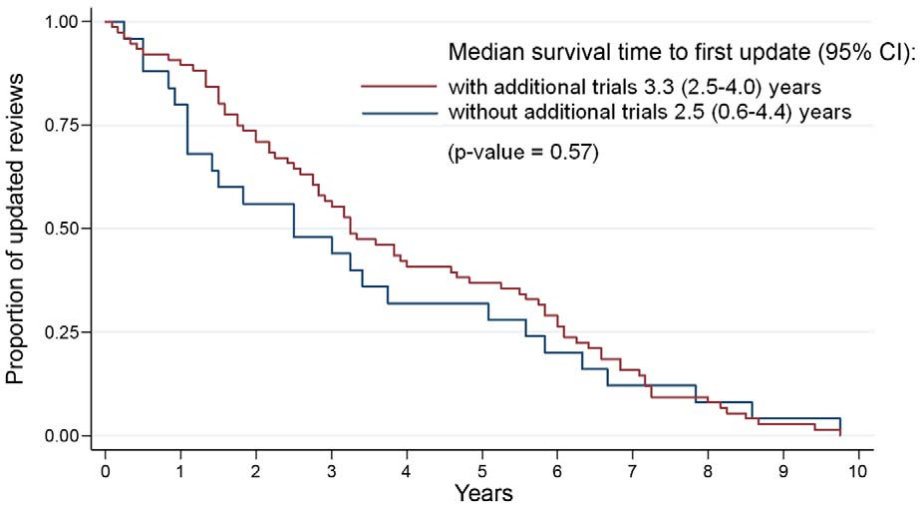
Kaplan–Meier curves for reviews with and without additional trials at first update.

For the 76 updated reviews that included new trials, the median time to first update was also 3.3 years (95% CI 2.5–4.0), as compared with 2.5 years (95% CI 0.6–4.4) in those 25 updated reviews that did not include new information (Fig. 3 and Table 2). This difference in time to update was not statistically significant (*p* = 0.57). Of the 76 updated reviews with additional trials, 10.5% (8/76) had been updated for the first time within 1 year, and 28.9% (22/76) within 2 years. Among updated reviews that included new trials, the fastest updates were seen in reviews of intrapartum issues (median 2.8 years; 95% CI 1.8–3.7). Among updated reviews that did not add new trials, the fastest updates were seen in those that concerned fetal complications (median 1.5 years; 95% CI 0.5–6.3). The reviews updated by the same original authors had the fastest time to update, with a median of 2.5 years (95% CI 1.8–3.2). The longest time to update was found in updated reviews by a new review team, with a median of 8.6 years (95% CI 4.2–13.0, Table 3).

**Table 3:** Time to first update of the 101 updated reviews, by review team.

	Reviews, n	Years to update, median (95% CI)
*Review team*		
Same original version	68	2.5 (1.8–3.2)
Included new author(s)	19	6.8 (6.1–7.5)
Changed authors(s)	11	5.8 (4.2–7.4)
New review team	3	8.6 (4.2–13.0)

### Quantitative changes at first update

In examining the details of updating (Table 4), we found that, of the 76 reviews that included additional trials, 25.0% (19/76) updated the original meta-analyses with new data and 28.9% (22/76) generated new comparisons. Both updated meta-analyses and new comparisons were present in 46% (35/76) of the updated reviews. Of the 76 reviews that included new trials, 71% (54/76) showed a quantitative change in the updated meta-analysis. The median of additional new trials was 1 (IQR 1–2 trials), and the median of increasing participants was 318 (IQR, 132–1193 participants).

**Table 4:** Characteristics of updated reviews that included additional trials at first update (76 reviews).

Characteristic	n (%)
Added new data to original meta-analysis	19 (25.0)
Made new comparisons	22 (28.9)
Added new trials to original meta-analysis and made new comparisons	35 (46.1)
Total	76 (100.0)

Of the 54 updated reviews that showed quantitative changes, the median change in the estimate of effect size was 18.2% (95% CI 13.2%–23.1%). Of these 54 reviews, 19 (35.2%), showed a change in effect size of between 10.0% and 24.9%. Only 9.3% (5/54) of reviews that presented risk ratios showed a change in the direction of the effect; in 2 reviews, these estimates changed from a protective to a risk effect, while in the other 3 the change was in the opposite direction. However, there was no change in the conclusions in comparison with those of the original reviews. The median change in 95% CI width for these 54 reviews was 30.8% (95% CI 19.4%–32.9%). A change in the 95% CI width of between 25% and 49.9% was seen in 33.3% (18/54), and a change in statistical significance was seen in 18.5% (10/54). Of these, the findings of 8 reviews changed from non-significance (*p*>0.05) to significance (*p*<0.05). Of the 54 reviews with quantitative changes, those with a higher number of participants than the original versions showed a greater than 50% change in effect size and in the width of the 95% CI. A similar degree of change was observed with respect to statistical change. Results and the interpretation of conclusions were affected in only 3.7% (2/54) of the reviews with quantitative changes. These 2 reviews had quantitative changes higher than 50%; the degree of change in effect size for these reviews was 53.8% and 62.9%, respectively, and of that of change in the width of the 95% CI, 76.5% and 88.7%, respectively. These 2 reviews also had the shortest time to first update among the 54 with quantitative changes: 1.08 and 1.58 years, respectively, whereas the median time to update among the other 52 was 3.3 years (95% CI 2.4–5.3).

### Predictive factors for time to first update

Factors that showed a statistically significant association with the time to first update as detected by univariate analysis were the inclusion of additional trials (HR 0.51; 95% CI 0.32–0.83), searching an additional database (HR 0.59; 95% CI 0.38–0.92), topic area in intrapartum issues (HR 1.86; 95% CI 1.03–3.37) and changed new review team (HR 0.20; 95% CI 0.6–0.66). These 4 factors were subsequently added to the Cox proportional hazard model. A shorter time to update was associated with the same original review team in the update (Table 5).

**Table 5:** Factors predicting time to first update of PCG reviews.

Factor	Adjusted HR (95% CI)
*Topic classification*	
Antenatal care	Reference
Pregnancy complications	0.56 (0.23–1.34)
Fetal complications	1.02 (0.45–2.28)
Intrapartum issues	1.55 (0.77–3.13)
Postpartum issues	2.12 (0.72–6.24)
Additional trials (≥3 trials; median)	0.61 (0.35–1.06)
Additional participants (≥576 participants; median)	1.92 (1.05–3.49)
Additional database	0.79 (0.44–1.41)
*Review team*	
Same original version	Reference
Included new author(s)	0.17 (0.79–0.38)
Changed author(s)	0.27 (0.12–0.61)
New review team	0.13 (0.03–0.73)

HR =  hazard ratio; CI  =  confidence interval.

## Discussion

Our study of a retrospective cohort of updated PCG reviews was conducted to ascertain the average time to update and factors associated with updating. The results showed a median time to first update of 3.3 years (95% CI 2.8–4.6). Only a third of reviews had undergone a first update within 2 years, the Cochrane Collaboration's recommended interval for the updating of reviews. Three quarters of the updated reviews (75%; 76/101) added new trials (a median of 3 trials, IQR 1–5 trials) and participants (a median of 576 participants, IQR 180–1386 participants). Among the updated reviews that included additional trials at the first update, 71% (54/76) showed a quantitative change in the updated meta-analyses. However, only 2 of those updates resulted in major quantitative changes that also altered the conclusions. A shorter time to update was associated with maintaining the original review team in the update.

The Cochrane Collaboration aims to support up-to-date, evidence-based decision-making in health care by regularly updating the systematic reviews in its database. An interval of 2 years after the initial publication has been recommended as an appropriate time before the first update. This enables Cochrane reviews to provide rigorous up-to-date evidence for decision-making in health care interventions. Our study showed that two thirds (68/101) of the PCG reviews published in 2007, Issue 3, had been updated after a longer interval than the recommended period of 2 years. This might reflect a low frequency of new trials in the areas of pregnancy and childbirth. Limited resources and competing time demands can also make it difficult for the members of a review group to carry out frequent updates.

Our results show that reviews involving a new author(s) or new review team in the update took longer to update (more than 5 years). This finding suggests that there are personnel and time constraints hindering the updating process that contribute to reviews being out of date. Similar results have been reported elsewhere [18]. Some reviews may have been conducted by individual researchers who may lack necessary resources and/or academic support for updating. By assisting in the updating process through developing strategies for monitoring the updating of reviews, reminding reviewers about updating, and supporting reviewers in the updating process, Cochrane review groups might help to keep the Cochrane database up to date.

For the 76 updated reviews in which new trials were added, our results indicate that the median time to update of the 22 reviews that searched an additional database was 5.8 years (95% CI 3.3–6.6), which was much longer than that for the 54 reviews with no new database, 2.9 years (95% CI 2.2–3.3). Perhaps this is an indication that the addition of information from a new database is time-consuming; however, there may be room for improvement in search strategies and the processing of information from new databases.

Our results showed details of updating times of reviews within each topic category. We found, in all topic categories, updating times were higher in reviews with additional new trials than in those without new trials. Additionally, the updating interval for reviews with additional new trials was greater than 2 years for all categories. We found that the time to update for reviews with additional new trials of intrapartum issues and postpartum issues was less than for other topic categories. However, these differences were not statistically significant, supporting our premise that trial development in the area of pregnancy and childbirth is slow.

Our results showed that the median time to the first update was 3.3 years (95% CI, 2.8–4.6). This interval is shorter than the 5.5 years (95% CI, 4.6–7.6 years) reported by Shojania and colleagues [11]. Our study was limited to Cochrane Pregnancy and Childbirth reviews, while the sample used by Shojania and colleagues (100 meta-analyses indexed in the *ACP Journal Club* from 1995 to 2005), was more general, and included only a minority (27%) of Cochrane reviews. Moreover, Shojania and colleagues identified the time by which reviews were out of date on the basis of qualitative signals that evidence is out of date. In the current study, we use the event of a revised publication to identify time to update, rather than identifying when reviews become out of date. If we had used Shojania and colleagues' criteria, the “survival time” found in our sample of PCG reviews would be have been longer. Only 33.3% of the updated reviews that showed a quantitative change (18/54) showed a change of 50% or greater, the cut-off used by Shojania and colleagues to identify reviews that had become out of date. By this criterion, the median survival time for reviews in our sample would have been 7.2 years (95% CI, 6.3–8.0). A previous study of CRs published from 1998, Issue 2, to 2002, Issue 2 indicated that 9% of updated reviews had revised conclusions [5]. Our study found that only 3.7% (2/54) of the updated reviews with quantitative changes also had revised conclusions. This might be because our sample was drawn from only 1 issue, and from only 1 of the 52 Cochrane review groups. However, the fact that the 2 reviews with revised conclusions showed quantitative changes of more than 50% in their meta-analysis of the primary outcome lends some support to the use by Shojania and colleagues of a 50% change as a cut-off value for an out-of-date review [11].

Our results show that, although 76 reviews had been updated with additional new trials, in 29% of these (22/76) no qualitative changes could be identified. This was because they did not add new data to the meta-analysis of original primary outcomes but, rather, added new comparisons to the updated version. From the 54 updated reviews that showed quantitative changes, around one fourth showed a change in effect size or in 95% CI width, and one fifth showed a change in statistical significance. A few reviews (5/54) showed a change in the direction of the estimate of risk ratios, but these changes were around 1 and still resulted in the same findings for statistical significance as the original meta-analyses. The highest percentage of change in effect sizes and 95% CI width were seen in updated reviews with the highest percentage of new trials and participants. Finally, we found only 2 reviews in which quantitative changes much greater than 50% led to altered conclusions. These results reflect a need to ascertain the optimal interval for the updating of PCG reviews. However, the recommended 2-year interval for updates should be re-evaluated to determine whether it is in fact appropriate in the area of PCG reviews.

There are limitations in our study: 1) we focused on systematic reviews that included meta-analyses. Our study does not address updating systematic reviews without meta-analyses or reviews for which there appears to be a robust evidence base which definitively answers questions about effectiveness and/or safety of an intervention. For reviews where there is already an ‘answer,’ it is unlikely that there will be new primary research and updating the systematic review may not be necessary. This view is in keeping with the Cochrane Collaboration's policy of maintaining current and up to date reviews. 2) We did not incorporate information on qualitative changes that were relevant to clinical contents. This was because of the difficulty in searching for this type of information within the study period. 3) We did not consider secondary outcomes because they were diverse and many have few studies contributing to a meta-analysis. Secondary outcomes are usually not as important as the primary outcome for answering the review question. 4) We did not assess changes in review methodology (i.e. in the Cochrane Handbook guidance) as a potential factor for updating. This was because of difficulty in identifying the Handbook guidance versions used by the review authors before 2007 Issue 3. Our study collected updated reviews that appeared before this time, and finally. 5) We focused only on a cohort of PCG reviews, and our findings might not be generalizable to reviews in other clinical areas, especially those in which the pace of new trials development is different.

Our study provides food for thought for those who produce, publish and use PCG reviews. Most of these reviews have been updated less frequently than the Cochrane updating policy recommendation stipulates. Very few updated reviews had changed conclusions, and those that did showed large quantitative changes. To ensure that PCG reviews are up to date, proactive strategies should be developed and implemented. Refinements to the Cochrane review guidelines could help to harmonize international standards in certain aspects of the updating process and minimize the waste of time and resources. Tools to identify an optimal interval between updates should be developed to help support well-informed decision-making in pregnancy and childbirth care.
